# Stroma‐derived extracellular vesicle mRNA signatures inform histological nature of prostate cancer

**DOI:** 10.1002/jev2.12150

**Published:** 2021-10-01

**Authors:** Alex P. Shephard, Peter Giles, Mariama Mbengue, Amr Alraies, Lisa K. Spary, Howard Kynaston, Mark J. Gurney, Juan M. Falcón‐Pérez, Félix Royo, Zsuzsanna Tabi, Dimitris Parthimos, Rachel J. Errington, Aled Clayton, Jason P. Webber

**Affiliations:** ^1^ Tissue Microenvironment Group Division of Cancer and Genetics School of Medicine Cardiff University Cardiff UK; ^2^ Wales Gene Park Henry Welcome Building Cardiff University Cardiff UK; ^3^ Wales Cancer Bank University Hospital of Wales Cardiff UK; ^4^ Section of Surgery, Division of Cancer and Genetics, School of Medicine Cardiff University Cardiff UK; ^5^ Department of Urology Cardiff and Vale University Health Board, University Hospital of Wales Cardiff UK; ^6^ Division of Infection and Immunity, School of Medicine Cardiff University Cardiff UK; ^7^ Exosomes Lab. CICbioGUNE‐BRTA Parque Tecnologico Derio Spain; ^8^ Centro de Investigación Biomédica en Red de enfermedades hepáticas y digestivas (CIBERehd) Madrid Spain; ^9^ IKERBASQUE Basque Foundation for Science Bilbao Spain; ^10^ Institute of Life Science Swansea University Medical School, Swansea University Swansea UK

**Keywords:** biomarker, extracellular vesicles, prostate cancer, RNA, stroma

## Abstract

Histological assessment of prostate cancer is the key diagnostic test and can predict disease outcome. This is however an invasive procedure that carries associated risks, hence non‐invasive assays to support the diagnostic pathway are much needed. A key feature of disease progression, and subsequent poor prognosis, is the presence of an altered stroma. Here we explored the utility of prostate stromal cell‐derived vesicles as indicators of an altered tumour environment. We compared vesicles from six donor‐matched pairs of adjacent‐normal versus disease‐associated primary stromal cultures. We identified 19 differentially expressed transcripts that discriminate disease from normal stromal extracellular vesicles (EVs). EVs isolated from patient serum were investigated for these putative disease‐discriminating mRNA. A set of transcripts including Caveolin‐1 (CAV1), TMP2, THBS1, and CTGF were found to be successful in discriminating clinically insignificant (Gleason = 6) disease from clinically significant (Gleason > 8) prostate cancer. Furthermore, correlation between transcript expression and progression‐free survival suggests that levels of these mRNA may predict disease outcome. Informed by a machine learning approach, combining measures of the five most informative EV‐associated mRNAs with PSA was shown to significantly improve assay sensitivity and specificity. An in‐silico model was produced, showcasing the superiority of this multi‐modal liquid biopsy compared to needle biopsy for predicting disease progression. This proof of concept highlights the utility of serum EV analytics as a companion diagnostic test with prognostic utility, which may obviate the need for biopsy.

## 1 INTRODUCTION

Prostate cancer presents a major healthcare burden with approximately 450,000 men in Europe, and 164,690 men in the USA, diagnosed each year (Rawla, 2019). Most men with prostate cancer will have slow‐growing, indolent, tumours. In a proportion of patients (10%–15%), however, the cancer will exhibit more aggressive behaviour with risk of nodal and bony metastases that leads to incurable disease. Early identification of men at risk of aggressive, as opposed to indolent, prostate cancer remains a major challenge. Whilst the diagnostic pathway can vary between different countries, diagnosis generally consists of digital prostate examination and assessment of levels of circulating prostate‐specific antigen (PSA), supported increasingly by multi‐parametric MRI. Elevated levels of PSA, can raise the suspicion of prostate cancer but a lack of sensitivity and specificity of such tests make it inadequate for prostate cancer diagnosis and differentiation between indolent and aggressive disease (Loeb et al., 2014). Furthermore, not all prostate cancer positive patients present with an elevated PSA, with a false negative rate approaching 30% for men with PSA < 4 ng/ml (Thompson et al., 2004). Accurate diagnosis, therefore, relies on biopsy and histological grading of the tissue based on Gleason score (Ahmed et al., 2017). A non‐invasive means for early identification of men with aggressive disease would be of high utility during prostate cancer diagnosis, with the potential to provide additional guidance when making decisions to undergo biopsy.

Many studies into the development of novel cancer biomarkers have focussed on features originating from cancer cells directly. The tumour microenvironment is, however, an integral component of cancer. It is widely appreciated that the stroma plays a critical role across the spectrum of solid malignancies (Ahmed et al., 2017; Mesker et al., 2009; Moorman et al., 2012). Interstitial stroma is extremely complex with a variety of activated‐resident cells and infiltrates that pro‐actively contribute towards tissue remodelling, immune evasion, treatment responsiveness, and disease progression. The importance of disease‐associated changes within the prostatic stroma was first highlighted over a decade ago (Ayala et al., 2003; Olumi et al., 1999; Tuxhorn et al., 2002), and high stroma to tumour ratios have since been associated with treatment resistance, and poor prognosis (Yanagisawa et al., 2007). The precise mechanistic processes by which stromal cells influence clinical outcome remain unclear.

Recently, researchers have begun to explore the role of nanometre‐sized extracellular vesicles as modulators of the tumour environment. We have previously demonstrated that extracellular vesicles, secreted from prostate cancer cells, can activate stromal cells causing them to take on a disease‐associated reactive stromal phenotype (J. Webber et al., 2010), which are capable of supporting tumour growth in preclinical murine models (J. P. Webber et al., 2015). Numerous characteristics that are specific to disease‐associated, but not normal, stromal cells have been identified following stimulation with prostate cancer extracellular vesicles (EVs) (J. P. Webber et al., 2016). Stromal cells themselves can also secrete EVs, whose exact properties and roles remain unclear, but it has been suggested these act to promote tumorigenesis supporting intercellular communication in a bi‐directional, reciprocal fashion (Josson et al., 2015).

EVs are a potential valuable source of biomarkers for various cancers (Dijkstra et al., 2014; Etayash et al., 2016; Sandfeld‐Paulsen et al., 2016), including prostate cancer (Del Re et al., 2017; Duijvesz et al., 2015), because they encapsulate multi‐omic constituents of the originating cell type, their secretion is elevated during tissue stress, and they are detectable in a range of body fluids. Whilst many studies have focussed on cancer EVs there has been little work exploring stroma‐derived EVs as biomarkers. Considering the strong correlation between stromal influence, Gleason grade and disease progression (Ayala et al., 2003), and the relatively high proportion of stromal cells to cancerous epithelia in aggressive cancers, we hypothesised that stroma‐derived vesicle‐associated markers may be useful as a surrogate measure of aggressive disease. Recently, cellular RNA from stromal cells has been shown to predict metastatic prostate cancer (Mo et al., 2017), and decreased mRNA expression of Caveolin‐1 (CAV1) within tumour‐associated stroma was linked to poor prognosis (Hammarsten et al., 2016).

Our current study presents a characterisation of normal vs disease‐associated prostate stromal cell EVs, highlighting several differences in the encapsulated EV RNA cargo. Some of these findings were recapitulated when analysing clinical specimens with several candidate mRNAs able to discriminate between patients with high (Gleason score ≥8) versus low Gleason score (Gleason score ≤6). Furthermore, our data show that EV‐mRNA can predict the probability of disease progression. Prediction of Gleason score, and disease progression, were further improved using a Random Forest machine learning‐based analysis, combining multiple EV‐mRNAs alongside PSA measurements. This approach provides a surrogate measure of histological scores, in a minimally invasive fashion. These data demonstrate the value of such multi‐modal measures informing a modelling tool that predicts likelihood of rapid progression. Analysis of serum EVs holds significant promise for identifying aggressive forms of prostate cancer and offering a companion tool to aid clinical decisions.

## 2 MATERIALS AND METHODS

### 2.1 Cell culture

Patient‐matched adjacent‐normal and tumour‐associated prostatic stromal cells, from a total of six patients, were obtained from the Wales Cancer Bank. Cells were isolated from radical prostatectomy cores, as previously described (J. P. Webber et al., 2016). Briefly, cells were isolated from radical retropubic prostatectomy cores, taken from sites of palpable disease and also from apparently normal tissue from the opposite side of the same prostate and subsequently maintained in DMEM/F12 media (Lonza, Wokingham, UK). The media were supplemented with 2 mM l‐glutamine (Lonza), 100 U/ml penicillin (Lonza) and 100 U/ml streptomycin (Lonza). Cell cultures were maintained in 10% FBS, depleted of bovine EVs by overnight ultracentrifugation at 100,000 × g (max) (using Quick Seal tubes; 70 Ti fixed angle rotor, κ‐factor of 44, Optima LE‐80K ultracentrifuge; Beckman Coulter, High Wycombe, UK) and serial filtration through 0.2 µm and then 0.1 µm vacuum filters (Millipore, Watford, UK). Cultures were confirmed free of epithelial cells by immuno‐fluorescence staining for Cytokeratins and used at passages 6–7.

### 2.2 Immuno‐fluorescent microscopy

Stromal cells were fixed with ice‐cold acetone:methanol (1:1) for 7 min, dried and blocked in 1% BSA (w/v) in PBS for 1 h. Cells were then stained with primary antibodies (1 µg/ml) against Vimentin (sc‐6260, Santa Cruz Biotechnology Inc, Heidelberg, Germany), Cytokeratin 8 (sc‐8020, Santa Cruz Biotechnology Inc), Desmin (sc‐58744, Santa Cruz Biotechnology Inc), or αSMA (sc‐32251, Santa Cruz Biotechnology Inc) for 1 h. Antibody detection was achieved using goat anti‐mouse IgG Fab’ Alexa488 conjugated (A‐11001, ThermoFisher Scientific, Paisley, UK) and nuclei stained with DAPI. Cells were viewed by wide‐field fluorescence and images were acquired on a Zeiss Axio Observer.Z1 system (Zeiss, Cambridge, UK), using a 20×/0.8 numerical aperture objective, run on Zen Pro software. Images shown are representative from ≥6 fields of view per biological triplicates.

### 2.3 Serum sample collection

Archived serum samples (1 ml per specimen) were provided by the Wales Cancer Bank (applications WCB 16/004 and WCB 21/008). Samples were included from men (a) attending participating Welsh NHS urology clinics (Velindre University NHS Trust, Aneurin Bevan University Health Board, Cardiff & Vale University Health Board and Swansea Bay University Health Board); (b) with known prostate cancer confirmed by biopsy and awaiting treatment; or (c) men previously treated for prostate cancer and presenting with disease progression or biochemical relapse. Samples were excluded from men (a) unable to give informed consent and/or (b) men attending Welsh NHS urology clinics where prostate cancer has not been mentioned or discussed. Following sample collection, and where possible, patients are followed up every year for the first 5 years and again at 10 years post‐treatment. After this, further, follow up only occurs if the patient presents with signs of disease progression or biochemical relapse.

Serum specimens were prepared from blood taken at initial diagnosis, obtained from patients with either low Gleason prostate cancer (Gleason score = 6) or high Gleason prostate cancer (Gleason score = 8–10). Briefly, blood samples were collected in serum separator tubes (Becton Dickinson, Wokingham, UK), and samples left to clot for 30 minutes at room temperature. Samples were then centrifuged at 3000 × g for 5 min, allowing the gel to form an interface between serum and the remainder of the sample. Serum samples were then transferred to sterile cryovials (500 µl aliquots) and stored at –80°C. Samples were labelled using pseudo‐anonymised barcodes. During initial investigation of candidate mRNA utility, we assessed mRNA expression within serum‐derived EVs from n = 20 total patients (n = 10 per group, comparing patients either with low Gleason or high Gleason prostate cancer). For candidate validation we assessed serum EVs from n = 40 patients (n = 20 per group, for patients with low Gleason or high Gleason prostate cancer). For purposes of assessing probability of progression‐free survival within the current study disease progression includes biochemical relapse, diagnosis of metastatic spread or cancer‐related mortality.

### 2.4 EV isolation

Stromal cell‐conditioned media was collected every five days, and EVs purified by filtration and ultracentrifugation, using established methods (Thery et al., 2006). Briefly, conditioned media were centrifuged twice at 400 × g for 6 m, then once at 2000 × g for 15 m, and filtered using a 0.2 µm syringe filter, to remove cells and debris. Supernatants were then centrifuged at 100,000 × g (max) (using Quick Seal tubes; 70 Ti rotor) for 2 h to pellet EVs, followed by a PBS wash at 100,000 × g (max) (using Quick Seal tubes; 70 Ti rotor) for 2 h. Isolation of EVs from cell‐conditioned media or patient serum (1 ml) by size exclusion chromatography was achieved using Exo‐spin™ midi columns (Cell Guidance Systems, Cambridge UK) (Welton et al., 2015). EV‐containing fractions were then pooled, and EVs isolated by centrifugation at 100,000 × g (using Quick Seal tubes; TLA‐110 fixed angle rotor, κ‐factor of 13, Optima Max‐XP ultracentrifuge; Beckman Coulter) for 2 h. EVs were resuspended in PBS, quantified using the micro‐BCA protein assay (ThermoFisher Scientific), and stored at –80°C.

### 2.5 Western blotting and immuno‐detection of EV‐associated proteins

Cell lysates were obtained using RIPA buffer (Santa Cruz Biotechnology Inc) supplemented with sodium orthovanadate, phenylmethylsulfonyl fluoride (PMSF) and protease inhibitor cocktail as per the manufacturer's instructions. Protein concentrations of cell and EV lysates were quantified by Bradford Assay, and equal amounts of protein (20 µg) loaded per well. Electrophoresis and immuno‐blotting were performed as previously described (J. Webber et al., 2014). Protein detection was achieved using primary antibodies (1 µg/ml) against ALIX (sc‐166952, Santa Cruz Biotechnology Inc), TSG101 (sc‐7964, Santa Cruz Biotechnology Inc), Vimentin (sc‐6260, Santa Cruz Biotechnology Inc), or GAPDH (Y3322, BioChain Institute Inc, CA, USA) prior to a goat anti‐mouse HRP‐conjugated secondary antibody (62‐6520, ThermoFisher Scientific) and ECL‐based detection of proteins using a C‐DiGit Blot Scanner (LI‐COR Biosciences UK Ltd, Cambridge, UK). Analysis of EV surface protein expression was performed using an established immuno‐phenotyping approach (J. Webber et al., 2014). Briefly, concentrated EV (1 µg/well), or fractions from size‐exclusion chromatography columns, were added to protein‐binding 96‐well plates (Greiner Bio‐One, Stonehouse, UK). Following overnight immobilisation of EVs, and blocking with 1% BSA (w/v) in PBS for 2 h at room temperature, bound material was labelled with primary antibodies (1 µg/ml) against CD9 (MAB1880, R&D Systems, Abingdon, UK), CD81 (MCA1847, AbD Serotec, Bio‐Rad Laboratories Ltd, Hertfordshire, UK), CD63 (MCA2142, Bio‐Rad Laboratories Ltd), Human Serum Albumin (HSA; 250 ng/ml; MAB1455, R&D Systems) or irrelevant isotype matched control immunoglobulins (14‐4714‐82, e‐bioscience, ThermoFisher Scientific) for 2 h at room temperature under gentle agitation. After three washes, goat anti‐mouse biotinylated antibody (diluted 1:2,500; NEF823001EA, Perkin Elmer, Buckinghamshire, UK) was added for 1.5 h at room temperature. After three washes, Europium‐conjugated streptavidin (diluted 1:1,000; Perkin Elmer) was added for 45 min. Finally, after six washes, specific signal was measured by time‐resolved fluorometry using a PHERAstar *FS* multilabel plate reader (BMG Labtech Ltd, Aylesbury, UK).

### 2.6 Cryo‐electron microscopy

Concentrated EV preparations were immobilised onto glow‐discharging holey carbon 200‐mesh copper grids (Quantifoil Micro Tools GmbH, Großlöbichau, Germany). Grids were vitrified using a Vitrobot (Maastricht Instruments BV, Maastricht, NL). Vitrified samples were imaged at liquid nitrogen temperature using a JEM‐2200FS/CR transmission cryo‐electron microscope (JEOL), as described previously (Yeung et al., 2018), with ≥6 microscopic fields assessed per sample.

### 2.7 Nanoparticle tracking analysis

Stromal cell‐conditioned media were corrected for differences in cell number at time of collection. Size and concentration of particles present within the cell‐conditioned media were assessed using the NanoSight™ nanoparticle tracking system (Malvern Instruments, Malvern, UK). Samples were diluted in particle‐free water (Fresenius Kabi, Runcorn, UK), as required, to obtain concentrations up to 2 × 10^9^ particles per ml within the specified linear range of the instrument. Analysis was performed on a NanoSight™ NS300 system with a 488 nm laser and temperature set to 25°C. Six videos of 60 s were taken in light scatter mode with controlled fluid flow with a pump speed set to 80. Videos were analysed using the batch analysis tool of NTA 3.1 software (version 3.1 build 3.1.54), where minimum particle size, track length and blur were set at “automatic.” The average area under the histogram from the six videos was used as a particle concentration measurement. Background measurements of culture media that had not been exposed to cells contained negligible particles.

### 2.8 RNA extraction and profiling

Total RNA from cells and isolated EVs was extracted using Tri‐Reagent (Sigma–Aldrich, Dorset, UK), as per the manufacturer's instructions. RNA samples were eluted in 11 µl of RNAse‐free water, aliquoted, and stored at –80°C. The size profile of isolated RNA was measured by capillary electrophoresis, using RNA 6000 Nano and Pico total RNA kits (Agilent Technologies UK Ltd, Cheshire, UK), in an Agilent Bioanalyzer 2100 instrument (Agilent Technologies UK Ltd). To assess purity of EV‐associated RNA, isolated EVs were incubated with proteinase K (0.05 µg/µl; Sigma–Aldrich) for 10 min at 37°C. Proteinase K activity was inhibited by the addition of PMSF (5 mM; Sigma–Aldrich) for 10  min at room temperature, followed by further incubation at 90°C for 5 min. EVs were then incubated with RNase A (0.5 µg/µl) for 20 min at 37°C to degrade unprotected RNA. For Control samples, PBS was added in place of proteinase K, PMSF, and RNase A. Prior to further analysis, RNA concentration was assessed by NanoDrop™ 2000 spectrophotometer (ThermoFisher Scientific), as per the manufacturer's instructions, and used to normalise sample input.

### 2.9 Reverse transcription and quantitative PCR (RT‐qPCR)

For initial profiling, culture‐derived stromal cell‐, and EV‐associated, RNAs samples (500 ng) were subjected to genomic DNA removal, cDNA synthesis, and pre‐amplification using the RT^2^ PCR System PreAMP kit (Qiagen, Manchester, UK) as per the manufacturer's instructions. Next, PAHS‐120Z Human Fibrosis RT^2^ Profiler PCR Arrays (SABiosciences, Qiagen) were used to analyse samples, following the manufacturer's protocol. Briefly, pre‐amplified cDNA from stromal cells, or their secreted EVs, were mixed with RT^2^ SYBR Green master mix and RNAse‐free water. The resultant PCR mix was distributed equally (25 µl/well) across the PCR array. The PCR amplification was performed in a StepOne Plus Real‐Time PCR System thermocycler (Applied Biosystems, ThermoFisher Scientific). Amplification was carried out by heating the samples to 95°C for 10 min, followed by repeating cycles of 95°C for 15 s and 60°C for 1 min (ramp rate = 1°C/sec), for a total of 40 cycles. Arrays contain inter‐plate and reverse transcription calibrators and the same cycle threshold was used for analysis of all arrays. To validate the expression of mRNA targets identified within the arrays, and to analyse EV‐associated mRNAs from patient serum, TaqMan gene expression assays (Applied Biosystems) were used. For these samples, reverse transcription was performed using a high‐capacity cDNA reverse transcription kit (Applied Biosystems), as described previously (J. P. Webber et al., 2015). Then, serum‐derived samples only were subjected to a targeted pre‐amplification using the TaqMan PreAmp master mix (Applied Biosystems) and associated TaqMan Gene Expression assays, as per the manufacturer's protocol, using an S1000 Thermal Cycler (Bio‐Rad Laboratories Ltd). Final qRT‐PCR analyses of samples were performed using TaqMan Gene Expression assays as described previously (J. P. Webber et al., 2015). The comparative CT method was used for relative quantification of target gene expression against that of a standard reference gene (GAPDH). Fold change of a specific target gene between samples was calculated as equal to 2^–∆∆CT^, and expression of a target gene relative to the reference gene calculated as equal to 2^–∆CT^, as described (Schmittgen & Livak, 2008).

### 2.10 Statistical analysis

Statistical analyses between experimental groups were performed using Prism‐4 software V4.03 (Graph Pad, San Diego, CA). Experiments with two experimental groups were evaluated using Mann–Whitney *U* test. In experiments with more than two experimental groups one‐way ANOVA with Tukey's post‐test was used. *P*‐values less than 0.05 are considered significant Graphs depict mean ± SE, from one representative experiment of at least three similar experiments, unless stated otherwise. To assess the use of multiple EV‐associated mRNAs in combination, for accurate prediction of histological grading of prostate cancers, a random forest model was built within R (software version 3.5.1) to classify our dataset into high *vs* low Gleason prostate cancer. Target gene expression was log2 transformed and feature selection was undertaken using the Boruta 6.0 library (Kursa & Rudnicki, 2010). Random decision forests were built using the randomForest 4.6‐14 library and explorations of forests performed using the randomForestExplainer 0.9 library (Liaw, 2002; Paluszynska & Jiang, 2019). The model was refined to minimise the out‐of‐box error estimates based on the 20‐sample training dataset, prior to validation using the 40‐sample test dataset. The specificity and sensitivity of EV‐associated mRNAs, as indicators of high *vs* low Gleason prostate cancer, were obtained using Receiving Operator Characteristic (ROC) curves. The Area under ROC curves (AUC) was used to compare the diagnostic performance of mRNAs and PSA, both individually and in combination. To assess probability of progression‐free survival (disease progression here includes biochemical relapse, diagnosis of metastatic spread or cancer‐related mortality) random forest survival models were built using randomForestSRC (2.8.0) on the training dataset before applying them to the test dataset.

## 3 RESULTS

### 3.1 EV secretion is enhanced in tumour activated stroma compared to adjacent, donor‐matched, normal stroma

**FIGURE 1:**
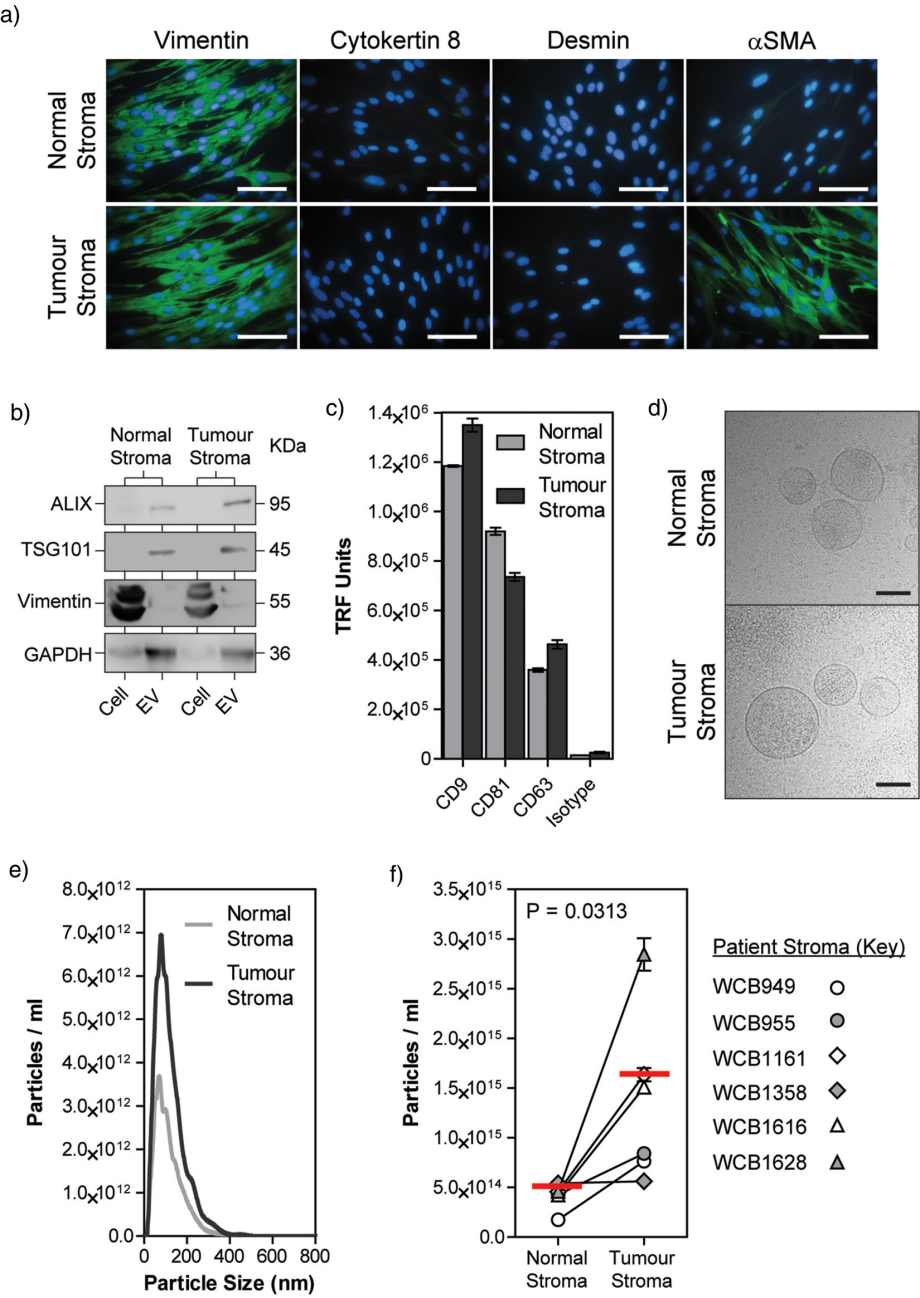
Secretion of extracellular vesicles is elevated in disease‐associated stromal cells. (a) Patient‐matched normal and tumour‐associated stromal cells, at passage 6, were seeded onto imaging chamber slides, and when 80% confluent subject to fixation and immuno‐fluorescent labelling for the specified markers (green). DAPI was used to identify the nucleus (blue) (Scale Bar = 100 µm). (b) Secreted EVs were isolated from stromal cell conditioned media by ultracentrifugation and compared to whole cell lysates by western blot. 20 µg of cell or EV lysates were loaded per lane. Membranes were probed using antibodies against ALIX, TSG101, Vimentin, or GAPDH. (c) Stromal cell‐derived EVs were immobilised onto plates and immunofluorescently labelled with antibodies against CD9, CD81, and CD63 (bars show mean ± SE, *n* = 3). (d) EVs isolated from normal or tumour‐associated stromal cell‐conditioned media were visualised by cryo‐electron microscopy (Scale Bar = 100 nm). Panels (a–d) represent contiguous data from patient WCB949 and is representative of six patient samples. (e) Size distribution of particles within preparations of stromal cell‐derived EVs, isolated by ultracentrifugation, was assessed by nanoparticle tracking analysis (data from patient WCB955 is shown, and is representative of six patient samples). (f) Subsequent assessment of concentration of particles within stromal EV isolates was assessed by nanoparticle tracking analysis and normalised based on cell number (mean ± SE, *n* = 6)

An aberrantly active stroma has long been associated with the growth and progression of prostatic tumours (Ayala et al., 2003; Olumi et al., 1999; Tuxhorn et al., 2002), and has since been linked to poor prognosis (Yanagisawa et al., 2007). Here, we compare secretion of extracellular vesicles from patient‐matched adjacent‐normal and tumour‐associated stromal cell pairs. Stromal cultures were established from fresh tissue (supplied by the Wales Cancer Bank), derived from radical prostatectomy specimens from six patients in which there was cancer in one half of the prostate but not the other (J. P. Webber et al., 2015). In agreement with our past studies (J. P. Webber et al., 2016), phenotypic analysis of stromal cultures by immuno‐fluorescent microscopy showed that both normal and tumour‐associated stromal cells have an elongated morphology that is typical of fibroblastic cells. Both cell types are highly positive for the mesenchymal marker Vimentin, but they lack epithelial Cytokeratins and the smooth muscle marker Desmin (Figure 1a). Tumour‐associated stromal cells could, however, be discriminated from normal stroma due to the presence of the myofibroblast marker, alpha‐smooth muscle actin (αSMA). The dual expression of Vimentin and αSMA observed within tumour‐associated stromal cultures, indicative of a myofibroblast‐like phenotype, is consistent with the stromal alterations seen in vivo and the accumulation of myofibroblasts (vimentin+ αSMA+) within the prostate interstitium (Tuxhorn et al., 2002).

Extracellular vesicles secreted from these stromal cells were isolated and subjected to a range of vesicle‐characterisation assays. First, we compared whole cell lysates with EV lysates by Western blotting. Upon matched loading of cell and EV lysates (20 µg/well), EVs showed enrichment of endolysosomal‐associated proteins; ALIX and TSG101 (Figure 1b). Conversely, Vimentin was present within cell lysates but barely detectable in EV lysates. Interestingly, whilst GAPDH was detectable in all samples, it was also enriched within EV lysates. Next, we assessed the expression of the tetraspanins CD9, CD81 and CD63 on the surface of stroma‐derived EVs. Both normal and tumour‐associated EVs were strongly positive for CD9, CD81 and CD63 (Figure 1c). In both EVs CD9 signal was greater than CD81, which was greater than that of CD63. Only minor differences in overall levels of tetraspanins were observed when comparing normal with disease EVs, whereby CD9 and CD63 appeared to increase, and CD81 decreased, in tumour‐stroma EVs.

Isolated EVs were also examined structurally by cryo‐electron microscopy, confirming similar unilamelar nano‐vesicles in each case, consistent with genuine isolation of small intact vesicles (Figure 1d). The size distribution profile (Figure 1e), of isolated EVs, from matched normal and tumour‐associated stromal cells, was assessed by nanoparticle tracking analysis (NTA). The mean diameter of particles was similar between these sources (104.37 and 109.37 nm, respectively). The concentration of particles present in cell‐conditioned media was also assessed by NTA (Figure 1f). Particle concentration was normalised for cell number, and the mean concentration of particles was 4.13 × 10^14^ particles/ml from normal stroma and 1.36 × 10^15^ particles/ml from tumour stroma, revealing a mean elevation of particle output by tumour‐associated stromal cells of approximately 3.3‐fold. Whilst the extent of this elevation varied between the six patient‐derived stromal samples, a significantly elevated concentration of particles was nonetheless observed in tumour‐associated stromal media from all six patient samples (Figure 1f).

These data highlight successful isolation of stromal cell‐derived extracellular vesicles, characterised in accordance with *the Minimal Information for Studies of Extracellular Vesicles (MISEV)* criteria (Lotvall et al., 2014; Thery et al., 2018), provided by the *International Society for Extracellular Vesicles*, and demonstrate that the secretion of EVs from tumour‐associated stromal cells is enhanced compared to normal stroma.

### 3.2 Enrichment of specific mRNAs within secreted EVs from tumour‐associated versus normal stroma

EVs are known to contain mRNAs, which can be delivered to recipient cells (Valadi et al., 2007). In addition, enrichment of specific mRNA within prostate cancer cell‐derived EVs has recently been reported (Lazaro‐Ibanez et al., 2017). Here we explored the mRNA cargo contained with prostatic stroma‐derived EVs by a variety of approaches to determine whether there are differences in the RNA cargo across the stromal EV types.

**FIGURE 2:**
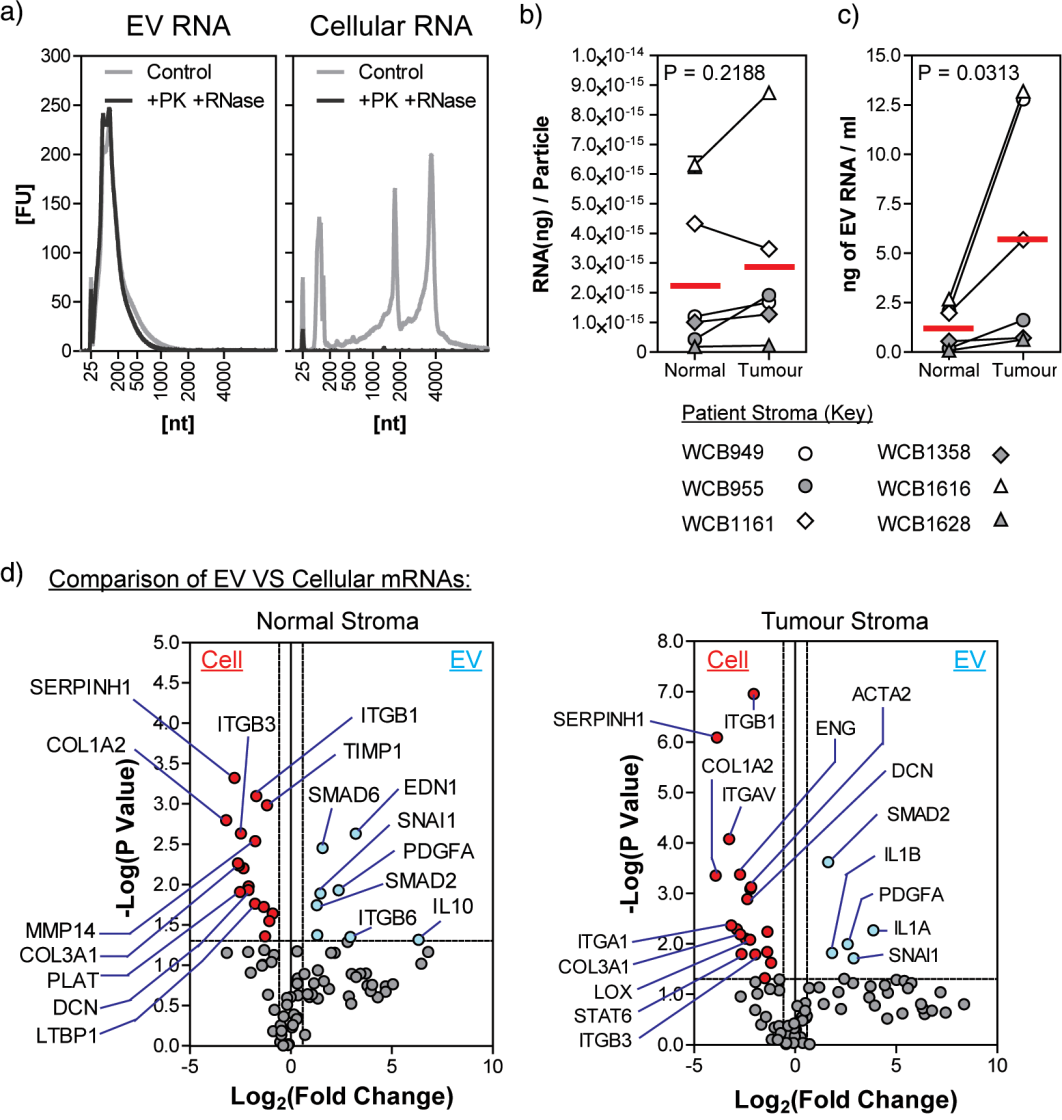
Stromal EVs are enriched for specific mRNAs. (a) Stromal EV‐associated RNA was isolated by phenol‐based extraction and quantified by NanoDrop™ 2000 spectrophotometer. Stromal EVs were incubated with proteinase K (0.05 µg/µl) and subsequently RNase A (0.5 µg/µl), or untreated (control), prior to assessment by bioanalyser to confirm presence of RNA within the lumen of EVs. Cellular RNA was used as control for the RNase activity (data shown is from patient WCB955 tumour stroma and is representative of six patient samples). Concentration of vesicular RNA was then determined (b) per particle and (c) per ml of cell‐number corrected cell‐conditioned media, from both normal and tumour‐associated stromal cells (*n* = 6 patients). (d) Volcano plots depicting results from RT^2^‐Profiler™ fibrosis PCR array, comparing cellular mRNAs with EV‐associated mRNAs, from normal stroma (left) or tumour‐associated stroma (right), revealing mRNAs enriched within cells (red dots) and mRNAs enriched within EVs (blue dots). Thresholds were applied at fold change ±1.5, and *P*‐value of ≤0.05 (students *t*‐test, from six patient samples)

Total RNA content from parental stromal cells and secreted EVs was assessed using an Agilent Bioanalyzer 2100 instrument. (Figure 2a). RNA profiles from stromal EVs were very different to those from parent cells. In agreement with previous studies, we have shown that EV‐associated RNA lacks both the 18S and 28S ribosomal RNA peaks (Lasser et al., 2012), which were both prominent in bioanalyser analysis of stromal cell‐derived RNA. Furthermore, the EV‐associated RNA was resistant to serial enzymatic degradation by proteinase K and RNAse A, demonstrating the absence of contaminating extra‐vesicular RNA. As a control for RNAse activity, cellular RNA was used (Figure 2a).

The concentration of EV‐associated RNA was assessed by NanoDrop™ and normalised to particle counts (obtained by NTA). We observed no statistically significant difference when comparing mean RNA concentration in normal stroma‐derived EVs (2.24 × 10^–15^ ng/particle) compared to EVs from patient‐matched tumour‐associated stroma (2.88 × 10^–15^ ng/particle) (Figure 2b). However, due to elevated secretion of EVs by tumour stroma, the quantity of EV‐associated RNA in the secretome is elevated in tumour‐associated stroma compared to that of normal stroma (5.77 and 1.26 ng/ml, respectively) (Figure 2c).

To explore the potential differential expression of mRNAs within disease versus normal stroma, we compared mRNA expression profiles of EVs and cells, from six patient‐matched pairs, using a qPCR array approach. This covers 84 mRNA transcripts known to be associated with stromal activation. Volcano plots show comparison mRNAs isolated from cells or EVs, from either normal or tumour stroma cultures (Figure 2d). When comparing cell versus EV mRNAs from normal stroma cultures, eight mRNAs were found to be at elevated levels within EVs whilst 17 mRNAs were more highly expressed within cells. Transcripts higher in normal stroma‐derived EVs include SMAD6, EDN1, IL‐10 and ITGB6, which would be consistent with regulatory/homeostatic function of the parent cells. When looking at RNA from tumour stroma cultures, five mRNAs were elevated within EVs whilst 18 mRNA within the cells. In the tumour stroma‐derived EVs, enrichment of IL1A, IL1B, and SNAI1 was consistent with a more inflammatory stromal phenotype typically associated with disease. Of the differentially elevated RNAs, there was approximately 52% overlap between mRNAs higher within normal versus tumour stroma cell‐derived RNA, whilst only 31% overlap between mRNAs higher within EV‐associated RNA. This suggests that differences in disease vs normal stroma are more exaggerated when evaluating EV‐RNAs rather than cellular‐RNAs, and may therefore provide a good surrogate measure of stromal cell status.

**FIGURE 3:**
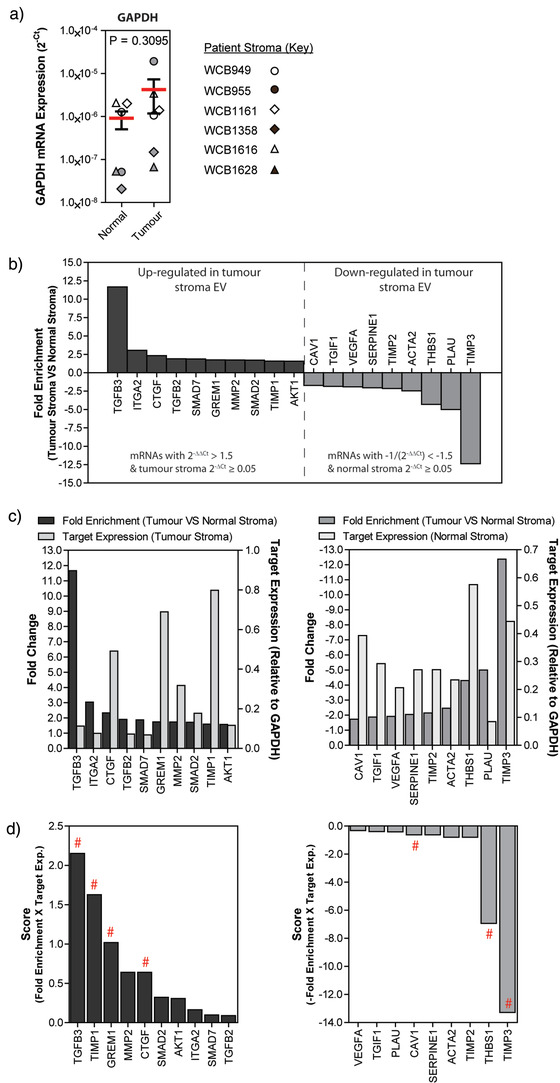
mRNAs are differentially expressed within tumour‐associated vs normal stromal EVs. (a) Expression of the housekeeping gene GAPDH in normal and tumour‐associated stromal cell‐derived EVs (*n* = 6 patients). (b) Waterfall plots depicting results from RT^2^‐Profiler™ fibrosis PCR array, showing 10 mRNAs that are up‐regulated, and nine mRNAs that are down‐regulated, in tumour‐stroma EVs (mean from six patients). (c) To identify mRNAs that were differentially expressed, yet also readily detectable, we applied thresholds of fold change ±1.5 (left axis) and expression relative to GAPDH, 2^–∆CT^ ≥0.05 (right axis) for mRNAs up‐regulated in tumour stroma EVs (left) or mRNAs down‐regulated in tumour stroma EVs (right). (d) A simple multiplication of fold change (2^–∆∆CT^) and target expression relative to GAPDH (2^–∆CT^) provided a means to identify mRNAs that may be either up‐regulated (left), or down‐regulated (right), yet also abundant within stromal EVs. Candidate mRNAs were selected from these plots for subsequent validation, as highlighted with #

Analysis of EV‐mRNA expression profiles, from all 6 stromal cell pairs, revealed no statistically significant difference in expression of GAPDH mRNA between tumour and normal stroma EVs (Figure 3a). Interestingly, however, differences in mRNA transcripts associated with stromal activation were observed. To confirm these were genuine expression differences and not changes due to PCR array technology, we selected candidates from the lists for downstream validation. For this, we included candidates with a ±1.5‐fold change in disease versus normal, with expression ≥5% (0.05) of that of the housekeeping gene, GAPDH (Figure 3b). We identified 10 mRNA targets that are elevated within EVs from tumour stroma. These included mRNA for TGFB3, ITGA2, CTGF, TGFB2, SMAD7, GREM1, MMP2, SMAD2, TIMP1, and AKT1. In addition, nine candidates comparatively reduced in tumour stroma EV were also identified; CAV1, TGIF1, VEGFA, SERPINE1, TIMP2, ACTA2, THBS1, PLAU, and TIMP3.

**FIGURE 4:**
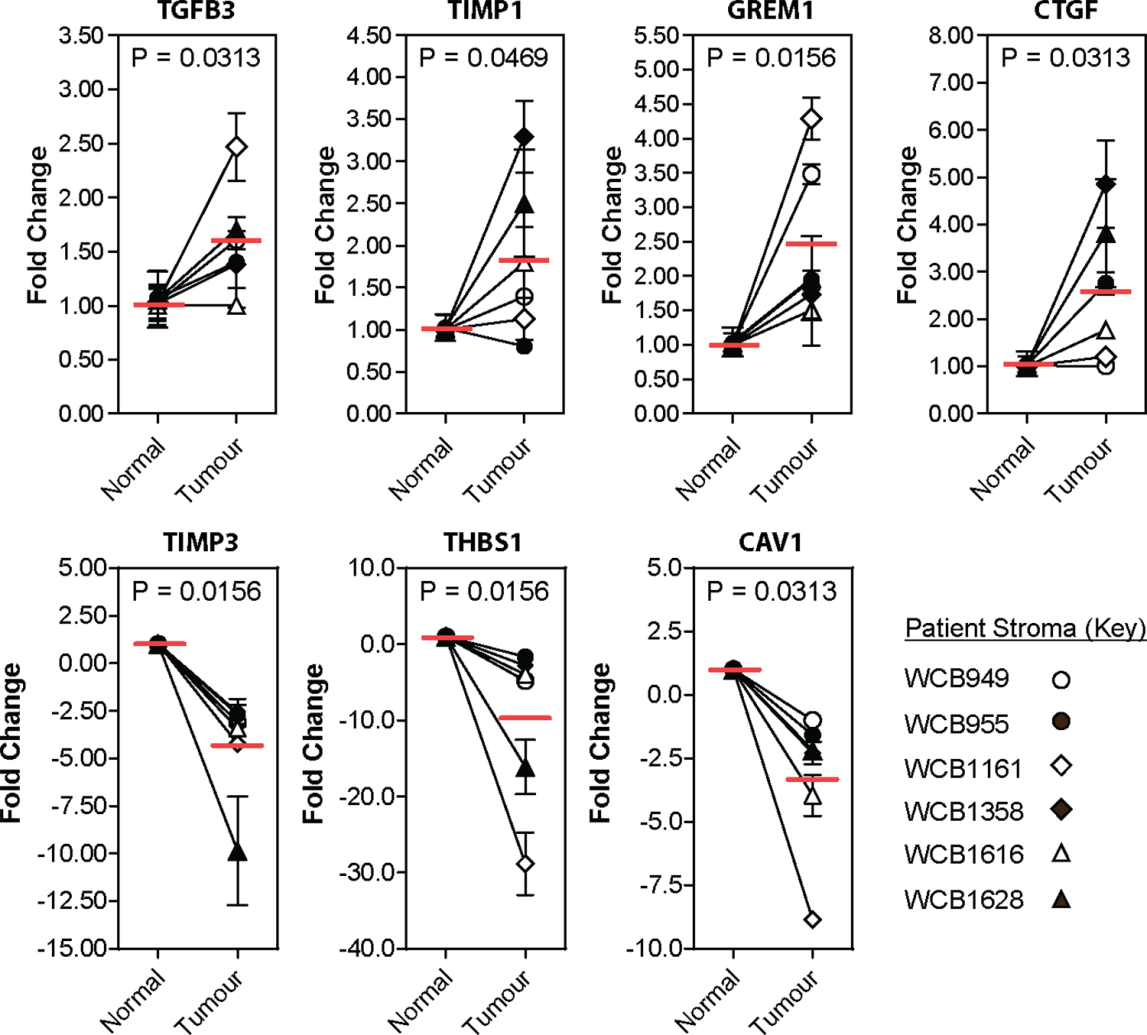
Validation of candidate stroma EV‐associated mRNAs. Candidate mRNAs, previously identified by RT^2^‐Profiler™ fibrosis PCR array, with differential expression between normal, and tumour‐associated, stroma‐derived EVs were validated by TaqMan gene expression assays (expression was normalised based on that of the house‐keeping gene, GAPDH). Candidate mRNAs predicted to be upregulated in tumour stroma EVs include TGFB3, TIMP1, GREM1 and CTGF. Whereas candidate mRNAs predicted to be downregulated in tumour stroma EVs include TIMP3, THBS1, and CAV1. Redline depicts mean candidate mRNA expression (*n* = 6 patient samples; statistical analysis was performed using Mann–Whitney *U* test)

To further assist choices for validation, we also considered the level of mRNA expression (in addition to differential expression as above). Plotting target expression (relative to GAPDH) alongside fold change (Figure 3c) highlight that some candidates, such as TIMP1, may have modest differential expression but high expression levels, and might be a readily measurable candidate for future biomarker studies. An mRNA “candidate score” was generated by multiplying fold enrichment and relative target expression. This was used to rank mRNAs of interest in EVs from either tumour or normal stromal cultures (Figure 3d). We then elected to analyse four EV‐mRNAs (TGFB3, TIMP1, GREM1, and CTGF) elevated in disease and three mRNAs (TIMP3, THBS1, and CAV1) relatively elevated in normal stroma EVs, using TaqMan gene expression assays (Figure 4). These data highlight relative expression of each mRNA target when comparing matched tumour versus normal stroma EVs from six patients. The trend in mRNA differences between tumour versus normal stroma EVs matches that seen with the array (Figure 3). The mean mRNA expression of TGFB3 (*P* = 0.0313), TIMP1 (*P* = 0.0469), GREM1 (*P* = 0.0156), and CTGF (*P* = 0.0313) were all increased in tumour stroma EVs compared to normal stroma EVs. Conversely, TIMP3 (*P* = 0.0156), THBS1 (*P* = 0.0156), and CAV1 (*P* = 0.0313) were all decreased in tumour stroma EVs compared to normal stroma EVs. The magnitude of fold change does not necessarily match that of the array, possibly highlighting differences between the two chemistries used. Nonetheless, these data successfully demonstrate differences between mRNAs expressed within EVs from tumour‐associated versus normal stroma.

### 3.3 Differential expression of serum‐derived EV‐associated mRNAs from patients with high versus low Gleason prostate cancer

Next, we explored whether these mRNA candidates were detectable in patient serum‐EVs, and if they had any value in assessing disease status within these individuals. In previous studies we have used an established size exclusion chromatography (SEC)—based approach for isolation of EVs from human serum (J. P. Webber & Clayton, 2013; Welton et al., 2015). Here we confirm successful separation of stroma vesicles from bulk soluble proteins present within stromal cell conditioned media using this approach (Supplemental Figure S1A). Conditioned media was fractionated, and a proportion of each fraction was directly coupled to protein‐binding plates and assessed for presence of EVs based on CD81 expression. This revealed a strong signal at fractions 8–12, in both normal and tumour stroma cell‐conditioned media (Supplemental Figure S1A, dark grey line). Total protein within fractions was also measured by BCA assay (Supplemental Figure S1A, pale grey line). Following successful isolation of EVs from cell‐conditioned media by SEC we used the same approach to isolate EVs from 1 ml of healthy donor serum (spiked with 100 µg of prostate cancer cell, Du145‐derived EVs) showing successful separation of EVs from >92% of the bulk soluble protein, represented by human serum albumin (HSA) (Supplemental Figure S1B). Resultant fractions were pooled into three groups, as shown, and quantification of RNA that was pelletable by ultracentrifugation (100,000 × g, for 2h) demonstrated that most RNA was present within the EV containing fractions (Supplemental Figure S1C). Importantly, detection of EV‐associated GAPDH mRNA was possible using TaqMan gene expression assays (Supplemental Figure S1D).

This methodology, therefore, was the basis of EV extraction from serum specimens, and mRNA analysis was performed on a total of 20 serum samples from men with prostate cancer. Among these, 10 patients were confirmed with low Gleason disease (Gleason score ≤6) and an equal number had clinically significant, high Gleason (Gleason score = 8–10) prostate cancer. The clinical information is summarised in Table 1.

**TABLE 1:** Patient‐derived serum samples

Low gleason score PCa patient samples							
			TNM classification				
Sample ID	Gleason score	Serum PSA (ng/ml)	Prefix^a^	T	N	M	Age (years)
1	3 + 3 = 6	6.3	P	T2c	N0	MX	67
2	3 + 3 = 6	4.6	P	T2c	N0	MX	57
3	3 + 3 = 6	5.6	P	T2c	N0	MX	66
4	3 + 3 = 6	6	–	–	–	–	65
5	3 + 3 = 6	8	–	–	–	–	58
6	3 + 3 = 6	2.8	P	T2b	N0	MX	48
7	3 + 3 = 6	5	P	–	–	–	52
8	3 + 3 = 6	6.1	P	TX	N0	MX	66
9	3 + 3 = 6	5.8	P	T2	NX	MX	56
10	3 + 3 = 6	5.2	P	T2	NX	MX	61

High gleason score PCa patient samples							
			TNM classification			
Sample ID	Gleason score	Serum PSA (ng/ml)	Prefix^a^	T	N	M	Age (years)
11	5 + 4 = 9	20.9	P	TX	NX	MX	58
12	4 + 5 = 9	7.9	P	T3	N0	MX	67
13	5 + 5 = 10	6.9	C	T4	N0	M0	78
14	3 + 5 = 8	11.7	P	–	NX	MX	71
15	4 + 5 = 9	4.5	P	T2	N0	MX	51
16	5 + 4 = 9	6.2	P	T3	N1	MX	68
17	4 + 5 = 9	26.3	P	T3	N0	MX	52
18	4 + 4 = 8	12.8	P	T3a	NX	MX	78
19	3 + 5 = 8	6.7	P	T2b	NX	–	66
20	4 + 5 = 9	5.8	P	T3b	N0	MX	64

^a^ Prefix abbreviations: C = TNM stage determined pre‐treatment; P = TNM stage determined following histological assessment of surgical specimen.

**FIGURE 5:**
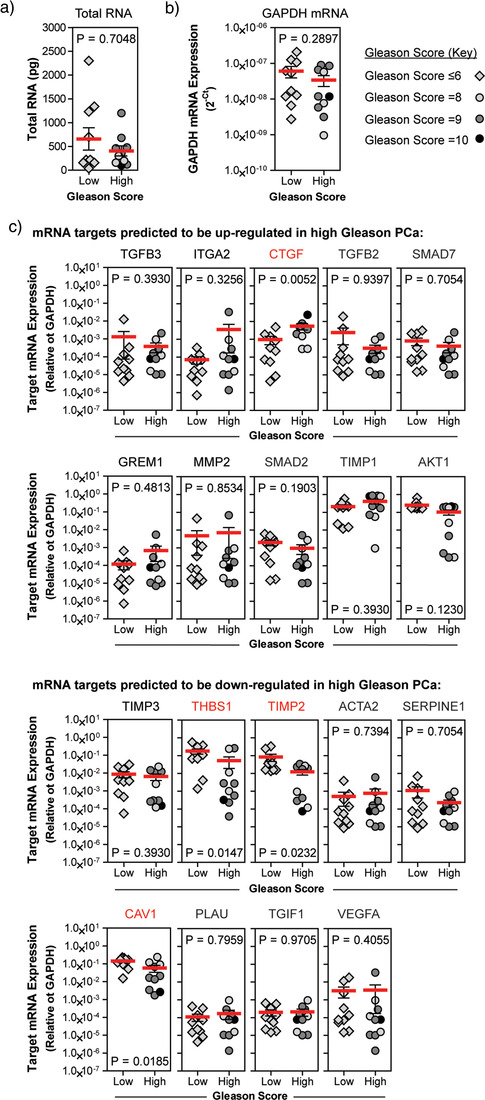
Expression of EV‐associated mRNAs show correlation with prostate cancer histology. EVs were isolated from serum obtained from patients with either low or high Gleason score prostate cancer by size‐exclusion chromatography. EV containing fractions were pooled, prior to EV pelleting by ultracentrifugation. (a) RNA was extracted from pelleted EVs, by phenol‐based extraction, and quantified by NanoDrop™ 2000 spectrophotometer. Reverse transcription was performed, along with pre‐amplification, prior to TaqMan gene expression assays to assess expression of (b) the housekeeping gene GAPDH and (c) the 19 mRNA targets previously identified within stromal EVs. Target mRNAs showing differential expression between low versus high Gleason score patient samples are highlighted in red. Red lines indicate mean ± SE target expression (*n* = 10 samples/group; statistical analysis was performed using Mann–Whitney *U* test)

Following quantification of total RNA within EVs, we observed no statistically significant difference in the amounts of vesicular RNA from each patient group (Figure 5a). To detect candidate mRNA, we first performed a pre‐amplification of the 19 mRNAs candidates and GAPDH. EV‐GAPDH mRNA expression did not differ between low or high Gleason patients (*P* = 0.2897) (Figure 5b). Of the 10 mRNAs that were enriched within cultured tumour‐stroma cell‐derived EVs, and therefore predicted to be elevated in patients with high Gleason prostate cancer, only CTGF mRNA expression was significantly elevated (*P* = 0.0052) (Figure 5c). Surprisingly mean expression of mRNA transcripts TGFB3, TGFB2, and SMAD7 appeared to be down‐regulated, however, these differences were not statistically significant. Of the nine mRNAs that were more abundant in normal stromal EVs, THBS1 (*P* = 0.0147), TIMP2 (*P* = 0.0232), and CAV1 (*P* = 0.0185) were significantly down regulated in high Gleason specimens (Figure 5c). In summary, by no means were all the cell culture‐generated candidates valuable in the context of clinical specimens, but some of these candidates may show utility in discriminating low from high Gleason prostate cancer patients (Figure 5).

**FIGURE 6:**
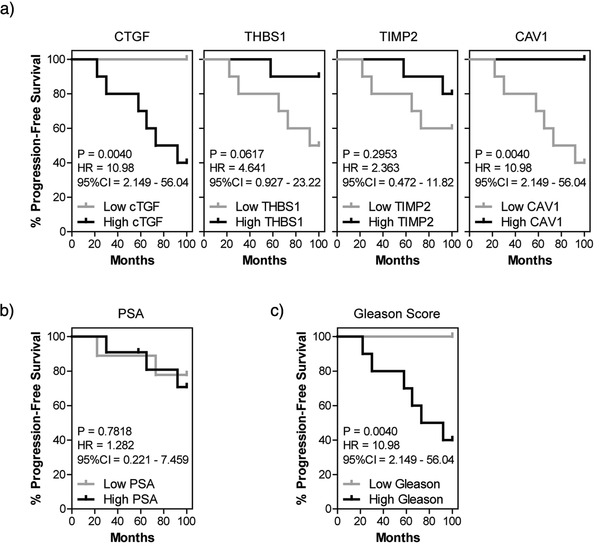
EV‐associated mRNAs have potential to predict disease progression. Kaplan–Meier curve analysis (log‐rank test) displaying progression‐free survival was performed on *(a)* EV‐associated mRNAs (CTGF, THBS1, TIMP2, and CAV1), (b) serum PSA, or (c) Gleason score (*n* = 20 patients)

We also considered the utility of EV‐mRNA transcripts in prediction of disease progression (Figure 6). Kaplan–Meier survival analysis was used to assess whether EV‐associated mRNAs could be used to predict the probability of progression‐free survival; where disease progression is defined here as biochemical relapse, diagnosis of metastatic spread or cancer‐related mortality. Focussing on the four mRNA transcripts that showed differential expression between high versus low Gleason patient‐derived EVs, we observed that either an increase in serum EV‐associated CTGF (HR = 10.98, *P* = 0.0040), or a decrease in EV‐associated CAV1 (HR = 10.98, *P* = 0.0040), correlate with an increased probability of disease progression. A decrease in either EV‐associated THBS1 (HR = 4.641, *P* = 0.0687) or TIMP2 (HR = 2.363, *P* = 0.2953) appeared to correlate with increased probability of disease progression, however these results were not statistically significant (Figure 6a). Importantly, serum PSA (cut‐off = 6.1 ng/ml; median PSA of this cohort) failed to predict disease progression (Figure 6b). Not only did the EV‐mRNAs outperform PSA, but EV‐associated CTGF and CAV1 were both shown to be just as good as Gleason score for prediction of disease progression (Figure 6c). Whilst this data was obtained from a small cohort of patients, and therefore requires further investigation, it nonetheless suggests that serum‐derived EV‐mRNAs could serve as a surrogate for biopsy during prostate cancer diagnosis.

### 3.4 Use of machine learning for prediction of histological severity and probability of disease progression

Receiver operating characteristic (ROC) curves were used to compare the utility of EV‐associated mRNAs in accurately discriminating patients with high versus low Gleason score prostate cancer (Supplemental Figure S2A). Assessment of EV‐associated CTGF, isolated from patient serum, revealed an area under the curve (AUC) of 0.8280 (95% CI = 0.6977–1.0220; *P* = 0.0065). Similarly, EV‐associated THBS1 gave an AUC of 0.8200 (95% CI = 0.6253–1.0150; *P* = 0.0156), TIMP2 gave an AUC of 0.8000 (95% CI = 0.5982–1.0020; *P* = 0.0234), and CAV1 an AUC of 0.8100 (95% CI = 0.6005–1.0200; *P* = 0.0192). These four EV‐mRNAs gave the best AUCs of the 19 mRNAs assessed (summarised in Table 2) and show good potential as classifiers for prediction of high versus low Gleason prostate cancer. The use of serum PSA as a prostate cancer biomarker has been examined extensively in previous studies, and past assessments of serum PSA as a prostate cancer biomarker have produced ROC AUCs ranging from 0.55 to 0.70 (Batra et al., 2014). Here, with this cohort of 20 individuals, we found PSA to be a good classifier in terms of predicting tumour histology, with an AUC of 0.8150 (95% CI = 0.6147–1.0150; *P* = 0.0173) (Supplemental Figure S2B). To improve the accuracy of EV‐associated mRNA predication of tumour histology, compared to PSA alone, we explored the use of AI‐based machine learning tools for combining multiple mRNA measures, potentially in addition to the PSA values.

**TABLE 2:** Results from Receiver Operating Characteristic (ROC) curve analysis of EV‐associated mRNA, or PSA, present within low versus high Gleason score prostate cancer patient serum

Target	AUC	95% CI	*P*‐value
TGFB3 mRNA	0.6200	0.3627–0.8773	0.3644
ITGA2 mRNA	0.6350	0.3841–0.8859	0.3075
CTGF mRNA	0.8600	0.6977–1.0220	0.0065
TGFB2 mRNA	0.5150	0.2487–0.7813	0.9097
SMAD7 mRNA	0.5550	0.2933–0.8167	0.6776
GREM1 mRNA	0.6000	0.3459–0.8541	0.4497
MMP2 mRNA	0.5300	0.2631–0.7969	0.8206
SMAD2 mRNA	0.6800	0.4365–0.9235	0.1737
TIMP1 mRNA	0.6200	0.3614–0.8786	0.3644
AKT1 mRNA	0.7100	0.4757–0.9443	0.1125
TIMP3 mRNA	0.6200	0.3600–0.8800	0.3644
THBS1 mRNA	0.8200	0.6253–1.0150	0.0156
TIMP2 mRNA	0.8000	0.5982–1.0020	0.0234
ACTA2 mRNA	0.5500	0.2866–0.8134	0.7055
SERPINE1 mRNA	0.5500	0.2906–08194	0.6776
CAV1 mRNA	0.8100	0.6005–1.0200	0.0192
PLAU mRNA	0.5400	0.2754–0.8046	0.7624
TGIF1 mRNA	0.5100	0.2446–0.7754	0.9397
VEGFA mRNA	0.6150	0.3590–0.8710	0.3847
PSA	0.8150	0.6147–1.0150	0.01729

**FIGURE 7:**
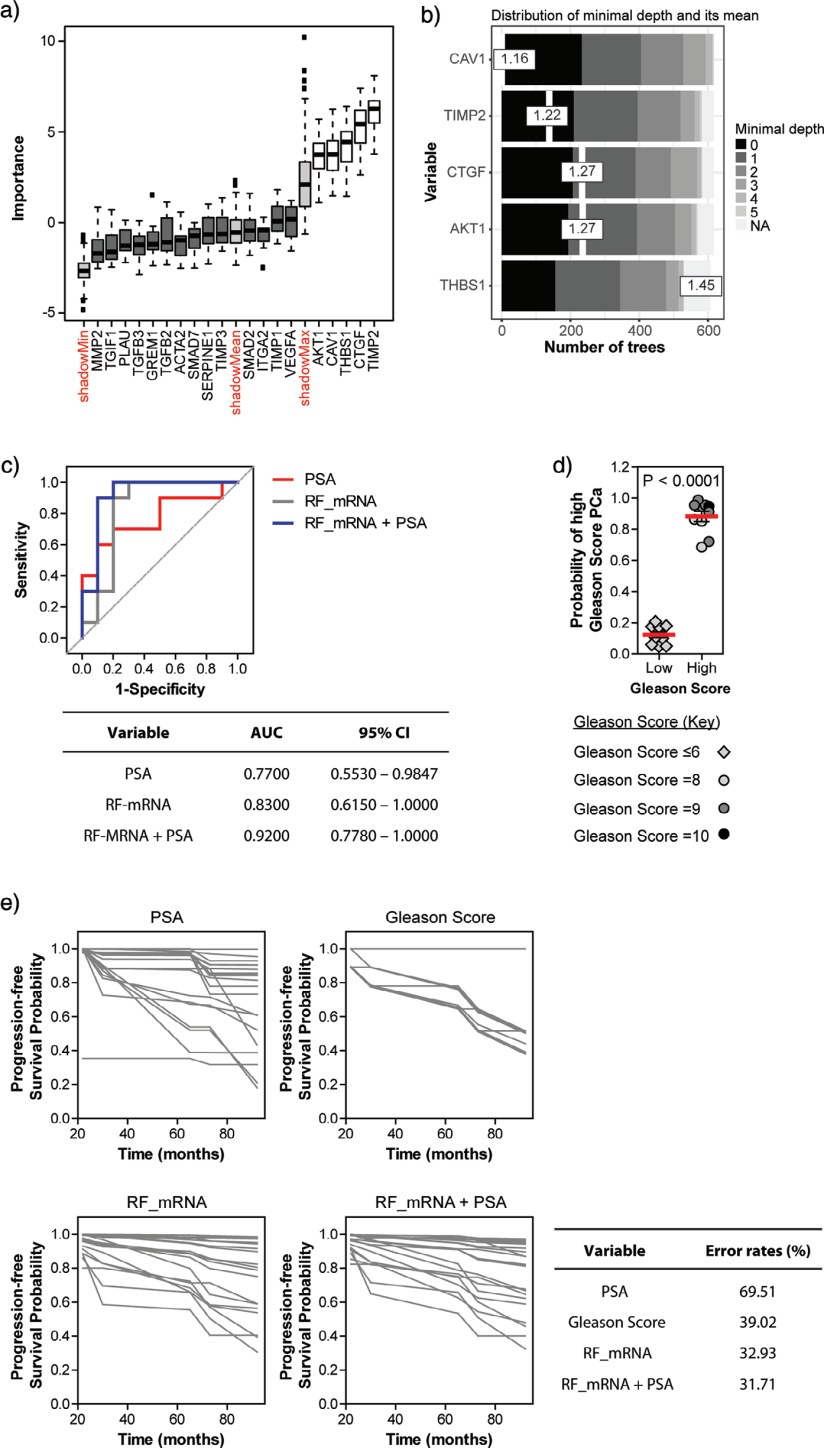
Biomarker accuracy is enhanced by combining multiple EV‐mRNAs. (a) Feature selection from mRNA expression data (log2 transformed), using Boruta 6.0 library in R, from EVs isolated from prostate cancer patient serum (*n* = 20). (b) mRNAs detected within serum‐derived EVs ranked based on the distribution of minimal depth during random forest formation. (c) Receiver operator characteristic (ROC) curve analysis of a selected combination of EV‐associated mRNAs (AKT1, CAV1, THBS1, CTGF, and TIMP2) (grey line), combined EV‐associated mRNAs with PSA (blue line), or PSA alone (red line) detectable within patient serum (*n* = 20) produced using the randomForest 4.6‐14 library and randomForestExplainer 0.9 library. (d) Successful prediction of high versus low Gleason score prostate cancer using the generated random forest model (*n* = 10 patients/group). (e) Random forest survival models, built using randomForestSRC (2.8.0), showing prediction of progression‐free survival based on serum PSA alone, Gleason score, random forest model from combined EV‐mRNAs, or the random forest model from combined EV‐mRNAs plus PSA. Each line represents survival of an individual patient (*n* = 20). Disease progression here includes biochemical relapse, diagnosis of metastatic spread or cancer‐related mortality

Following log2 transformation of EV‐mRNA expression data, Boruta feature selection performed in R was used to identify attributes of greatest importance in terms of discriminating high *vs* low Gleason prostate cancer patients. EV‐mRNAs deemed to be of genuine importance (above the “shadowMax”) in discriminating the two patient groups include AKT1, CAV1, THBS1, CTGF, and TIMP2 (Figure 7a). With the addition of AKT1, this agrees with our previous data showing that serum‐derived EV‐mRNAs CAV1, THBS1, CTGF, and TIMP2 are differentially expressed when comparing patients with either high or low Gleason disease (Figure 5c). The selected EV‐mRNAs can be ranked in terms of importance, based on the distribution of minimal depth, during random forest formation (Figure 7b). As the mean minimal depth of the five EV‐mRNAs is very close, ranging from 1.16 to 1.45, we can assume that they are of comparable importance for splitting the data set into patient groups. Subsequent use of a random forest algorithm to assess the utility of these five EV‐associated mRNAs in combination to discriminate patients with high versus low Gleason score prostate cancer produced a ROC AUC of 0.83 (95% CI = 0.6150–1.0000), which is markedly greater than the AUC of 0.7700 (95% CI = 0.5530–0.9847) obtained for serum PSA, in the same programme (Figure 7c). Interestingly, if we combine the five EV‐associated mRNAs, with PSA, the AUC is increased further to 0.9200 (95% CI = 0.7780–1.0000), suggesting that this is the best combination of markers assessed for separating patients with high Gleason score prostate cancer from those with low Gleason disease. The combination of these five EV‐mRNAs and PSA measurements show great potential for accurately identifying patients with high Gleason score, and hence clinically significant prostate cancer in a manner that does not require invasive tissue sampling by biopsy (Figure 7d).

We assessed if a machine‐learning approach could generate a predictive model for disease progression, based on various inputs. Progression‐free survival for each patient was predicted based on serum PSA alone or combined expression of five EV‐associated mRNAs or the five EV‐mRNAs with PSA (Figure 7e). Out‐of‐bag error rates from the random forest model indicate an error of 69.51% when predicting progressive disease if based on PSA measurements alone. This indicates the predication is correct only in 30% of cases. The error rate decreases to 32.93% when using a combination of EV‐mRNAs AKT1, CAV1, THBS1, CTGF and TIMP2, and is further slightly decreased to 31.71% error (68.29% accuracy) when combining EV‐mRNAs with PSA. Interestingly, this error rate is less than that observed when predicting progression‐free survival using Gleason score alone, which equalled 39.02%, and this latter measurement requires surgical biopsy and histopathological assessment.

### 3.5 Validation of EV‐associated mRNAs as predictors of histological severity and disease progression

**FIGURE 8:**
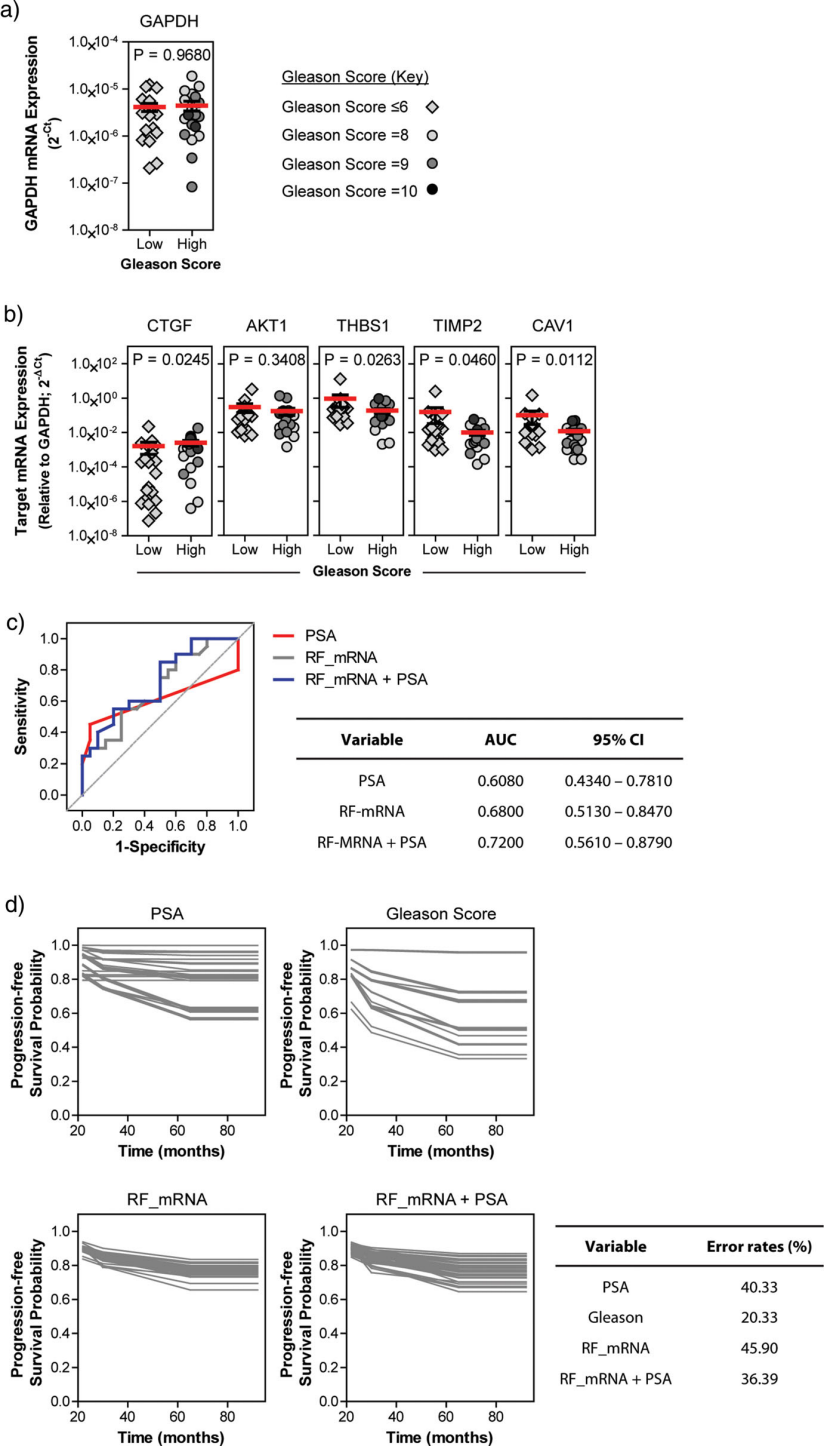
Validation of EV‐associated mRNAs as predictors of prostate cancer histology and risk of disease progression. RNA was extracted from serum EVs, from an independent test cohort of patients with low or high Gleason score prostate cancer. Reverse transcription, and subsequent pre‐amplification, prior to TaqMan gene expression assays were used to assess expression of (a) GAPDH and (b) the five mRNA targets (AKT1, CAV1, THBS1, CTGF, and TIMP2) previously shown to be differentially expressed in patient serum. Red lines indicate mean ± SE target expression (*n* = 20 samples/group; statistical analysis was performed using Mann–Whitney *U* test). (c) Receiver operator characteristic (ROC) curve analysis of a selected combination of EV‐associated mRNAs (AKT1, CAV1, THBS1, CTGF, and TIMP2) (grey line), combined EV‐associated mRNAs with PSA (blue line), or PSA alone (red line) generated by testing the random forest model on the test cohort of serum samples (*n* = 40). (d) Random forest survival models for predicting risk of disease progression, applied to the validation cohort, showing probability of progression‐free survival based on serum PSA alone, Gleason score, combined EV‐mRNAs, or the combined EV‐mRNAs plus PSA. Each line represents survival of an individual patient (*n* = 40). Disease progression here includes biochemical relapse, diagnosis of metastatic spread or cancer‐related mortality

To confirm the potential of EV‐associated mRNAs as indicators of histological severity and disease progression we sought to validate our findings on an independent cohort of 40 serum samples from men with prostate cancer. Of these, 20 patients were confirmed with low Gleason disease (Gleason score ≤6) and 20 had clinically significant, high Gleason (Gleason score = 8–10) prostate cancer. The clinical information is summarised in Table 3. Following quantification of total RNA within isolated EVs, and pre‐amplification of mRNA candidates, the expression of GAPDH, CTGF, AKT1, 1, TIMP2, and CAV1 were examined by TaqMan gene expression assays. The expression of EV‐associated GAPDH mRNA did not differ between low or high Gleason patients (*P* = 0.9680) (Figure 8a). Expression of each of the 5 mRNA candidates (Figure 8b) followed similar patterns to those of the initial sample set. CTGF mRNA expression was significantly elevated (*P* = 0.0245) in patients with high Gleason disease compared to those with low Gleason prostate cancer. AKT1 showed no significant difference (*P* = 0.3408). THBS1 (*P* = 0.0263), TIMP2 (P = 0.0460), and CAV1 (*P* = 0.0112) were all significantly down‐regulated in high Gleason specimens.

**TABLE 3:** Patient‐derived serum samples used for assay validation

Low gleason score PCa patient samples							
			TNM classification				
Sample ID	Gleason score	Serum PSA (ng/ml)	Prefix^a^	T	N	M	Age (years)
21	3 + 3 = 6	5.6	–	–	–	–	56
22	3 + 3 = 6	8.8	–	–	–	–	61
23	3 + 3 = 6	10.8	–	–	–	–	67
24	3 + 3 = 6	6.1	–	–	–	–	69
25	3 + 3 = 6	2.3	–	–	–	–	63
26	3 + 3 = 6	6.3	P	T2c	NX	MX	71
27	3 + 3 = 6	5.8	P	T3a	NX	MX	60
28	3 + 3 = 6	7.4	P	T2c	NX	MX	61
29	3 + 3 = 6	10.1	P	T2	NX	MX	65
30	3 + 3 = 6	8.8	P	T2a	NX	MX	67
31	3 + 3 = 6	6.5	P	T2c	NX	MX	57
32	3 + 3 = 6	2.8	P	T2	NX	MX	56
33	3 + 3 = 6	2.8	C	T3a	NX	MX	57
34	3 + 3 = 6	6.5	P	T2	NX	MX	40
35	3 + 3 = 6	3.2	P	T2	NX	MX	49
36	3 + 3 = 6	12.7	P	T3	N0	M0	84
37	3 + 3 = 6	8.2	P	T3a	NX	MX	55
38	3 + 3 = 6	10.8	P	T3a	NX	MX	58
39	3 + 3 = 6	7.1	P	T3a	N0	MX	49
40	3 + 3 = 6	18	P	T3a	N0	–	64

High gleason score PCa patient samples							
			TNM classification				
Sample ID	Gleason score	Serum PSA (ng/ml)	Prefix^a^	T	N	M	Age (years)
41	3 + 5 = 8	8.2	P	T3a	N0	MX	68
42	4 + 5 = 9	8.5	C	T2b	NX	MX	73
43	5 + 5 = 10	12.7	P	T3	N0	M0	84
44	3 + 5 = 8	22.9	C	T2b	N0	M0	73
45	3 + 5 = 8	71	C	T3	NX	M1	86
46	4 + 4 = 8	41	C	T2b	N0	M0	67
47	3 + 5 = 8	11	P	T3a	N0	MX	66
48	4 + 4 = 8	6.9	P	T3a	N0	MX	52
49	4 + 5 = 9	19.6	P	T3b	N0	MX	66
50	4 + 4 = 8	32.4	P	T3b	N1	MX	82
51	4 + 5 = 9	29.2	P	T3a	N1	MX	64
52	4 + 5 = 9	18.5	P	T3a	N0	MX	73
53	4 + 5 = 9	8.2	P	T2a	N0	MX	67
54	3 + 5 = 8	26.6	P	T3b	N0	MX	58
55	3 + 5 = 8	3.8	P	T2	N0	MX	61
56	5 + 4 = 9	603	C	T4	N1	M1	71
57	4 + 5 = 9	126.5	C	T2a	NX	M1	72
58	4 + 4 = 8	42	P	T2c	N1	M0	58
59	4 + 5 = 9	1.4	C	T3b	N0	MX	90
60	5 + 5 = 10	19.9	C	T3	NX	M‐	83

^a^ Prefix abbreviations: C = TNM stage determined pre‐treatment; P = TNM stage determined following histological assessment of surgical specimen.

Within this biospecimen cohort, the random forest model, created from the training dataset, showed that the five EV‐associated mRNAs in combination could again discriminate patients with high vs low Gleason score prostate cancer producing a ROC AUC of 0.6800 (95% CI = 0.5130–0.8470), which is greater than the AUC of 0.6080 (95% CI = 0.4340–0.7810) obtained for serum PSA (Figure 8c). However, as was observed in the training data set, the AUC is increased further, to 0.7200 (95% CI = 0.5610–0.8790), by combining the five EV‐associated mRNAs with PSA. This is comparable to a similar study exploring utility of urine‐derived EV‐RNAs for prostate cancer diagnosis (Connell et al., 2019) and highlights that use of the EV‐mRNAs alongside PSA provides enormous potential for predicting prostate tumour histology in a non‐invasive manner.

Finally, we tested our progression‐free survival prediction model on the validation cohort (Figure 8d). Here, out‐of‐bag error rates from the random forest model indicate an error of 40.33% when predicting progressive disease if based on PSA measurements alone. The error rate for the five EV‐mRNAs in combination was 45.90%, which decreased to 36.39% error (63.61% accuracy), when using a combination of EV‐mRNAs with PSA. Unlike for our training dataset, this error was higher than that observed when using Gleason score alone, which equalled 20.33%. Nonetheless, the data indicate that use of EV‐associated mRNAs alongside PSA can increase accuracy when predicting progression‐free survival by non‐invasive methods, yet there is potential to improve the predictive value by further explorations of stromal features of serum EVs.

In summary, sampling serum for the specified EV‐mRNA alongside PSA shows potential to estimate the probability of progression without the need for histological assessment. This approach or future variant based on this concept may provide a highly useful modality for diagnosing aggressive prostate cancer, aiding clinical decision making around biopsy and other interventions.

## 4 DISCUSSION

There remains an unmet need for development of non‐invasive biomarkers for early diagnosis of patients with clinically significant prostate cancer, and there has been much interest in the use of EVs (Del Re et al., 2017; Duijvesz et al., 2015). Compared to other biomarkers such as proteins (e.g., PSA) and circulating tumour cells (CTCs), EVs offer numerous advantages including abundance and stability (Shao et al., 2015), and have recently been utilised in development of diagnostic assays (McKiernan et al., 2016). Transition from early neoplastic lesions through to aggressive forms of prostate cancer has been linked to disease‐associated changes within the prostatic stroma (Yanagisawa et al., 2007) and hence we sought to evaluate circulating EVs carrying features of altered stroma as potential indicators of dangerous, as opposed to indolent, disease.

Our comparison of in vitro stromal cell‐derived EVs revealed little difference in morphology between normal and disease‐associated stromal EVs. However, an escalation of EVs present within conditioned media from tumour stromal cells was observed. The net effect, therefore, in terms of detecting these in a clinical setting, is that we may expect more stromal EV to be available in disease compared to non‐disease situations. When evaluating RNA content of EVs, whilst there was no difference in the RNA amount per vesicle, the net effect of heightened EV output means that tumour stromal cells secrete more RNA via the release of EVs. In a cancerous microenvironment, hypercellularity due to an expanding epithelial cell population and infiltrates are also likely to contribute to the net composition of circulating EVs. As yet, the relative contributions that stromal cell EVs make to this overall altered pool has not been quantified. In settings where the stromal cell to tumour cell ratio is elevated, the evaluation of stromal EVs as disease indicators may be very appropriate.

Compositional differences observed between cellular and vesicular RNAs allude to a sorting mechanism capable of regulating the loading of EV with distinct RNA cargoes, and is consistent with other studies comparing EV and cellular RNAs (Hinger et al., 2018; Szostak et al., 2014). Importantly, we identified several mRNAs with an altered expression when we compared EVs from normal stroma to tumour stroma‐derived EVs. Many of these mRNAs have previously been linked to disease‐associated changes within the surrounding stroma (Hammarsten et al., 2016; Mammadova‐Bach et al., 2018; Reinertsen et al., 2012; Yang et al., 2005). Yet to our knowledge, and following exploration of the TCGA PanCancer Atlas dataset available via cBioportal (Cerami et al., 2012; Gao et al., 2013), an altered expression of AKT1, CAV1, THBS1, or TIMP2 has not been previously associated with progression to aggressive prostate cancer. The exception to this is CTGF, which has previously been associated with aggressive disease (Kim et al., 2020).

An advantage of our study is the use of a PCR array focussed on stroma‐related transcripts. This provides a read‐out most likely to identify disease‐related alterations that are indicative of interstitial tissue remodelling, as a feature of tissue response to neoplastic injury. Whilst the array is focussed to known elements responsible for directing and regulating interstitial environments, it is also limited in transcript coverage. Having provided conceptual proof, a more extensive RNA‐profiling of stromal EVs is now clearly warranted. Our unpublished studies employing RNA sequencing reveal that coding RNA represents a minor proportion (<5%) of the total RNA. Hence, there is justification for wholesale RNA analysis of stromal EV to map differentially expressed non‐coding RNA, and changes associated with clinically significant disease.

A significant challenge impeding the use of EVs as disease biomarkers is their isolation and detection from complex biofluids. There have been advances in recent years, including protocols for separation of EVs from contaminating soluble protein aggregates and lipoproteins (Karimi et al., 2018). Our chosen approach, reliant on SEC followed by an ultracentrifuge step for isolating EV from serum specimens is imperfect, as it does not eliminate such non‐vesicular particulates, and the isolate will of course be replete with EV from a diverse range of cellular sources such as platelets. When dealing with complex input material the isolation and detection of EVs from a specific cell‐type of origin remains a challenge. Here, by using low passage patient‐derived stromal cultures, we intentionally simplified the vesicle source for marker discovery allowing a clear definition of disease versus non‐disease stromal phenotypes. Our in vitro studies highlighted 10 mRNAs that were elevated, and nine mRNAs downregulated, in tumour stroma EVs compared to normal stroma EVs. We hypothesised that the differentially expressed EV‐associated mRNAs could identify patients with a higher proportion of tumour reactive stroma, and therefore predict histological nature of disease. Of the 19 mRNAs tested, CTGF, THBS1, TIMP2, and CAV1 were confirmed as differentially expressed in a small clinical cohort, and subsequently validated in an independent cohort. Given the complexity of serum derived EV isolates, it is perhaps unsurprising that not all of the 19 mRNA candidates of the culture system were relevant in clinical samples. These EV‐associated mRNAs were shown to have some utility in distinguishing patients with high Gleason score disease from those with a low Gleason score, and therefore clinically significant from insignificant disease. Failure of the remaining 14 mRNAs to differentiate disease status is likely due to the repertoire of EVs from a diverse assortment of cell types present within the serum, impeding detection of less abundant stroma‐derived EV‐associated mRNAs. Whilst further studies are required to refine detection of tumour stroma‐derived EVs in serum, the current study provides proof‐of‐concept evidence that this is a viable approach with surprising power for predicting histological status.

Many patients with prostate cancer have slow‐growing indolent tumours, but a proportion of patients have aggressive tumours that require radical treatment and there is need for better tests to enable patient stratification. Previous assessment of PSA as a prostate cancer biomarker has yielded mixed results, but in general PSA is not considered to hold predictive value (Batra et al., 2014). We used a random forest‐based approach to identify a specific combination of five mRNAs (CAV1, THBS1, CTGF and TIMP2 and AKT1) that could successfully discriminate between patients with high versus low Gleason score, with greater accuracy than PSA. This initial investigation was, however, completed using serum samples from just 20 patients, and caution is required due to the bootstrapping approach used and the potential risk of over‐fitting the data. Consequently, our random forest model was tested using an independent validation cohort of 40 serum samples. Further development and refinement of such an assay will, however, require larger sample cohorts, from which more substantive training and test datasets could be used to eliminate the potential issues arising from small datasets, whilst providing further validation for the machine learning approach. Nonetheless, machine learning is a valuable tool for utilisation when considering multiple diagnostic features (Lekchnov et al., 2018; Tuo et al., 2018). Here, we demonstrate that EV‐mRNAs could predict the risk of disease progression with greater accuracy than that achieved by PSA alone, but the predictive accuracy of combining these measurements (63.61% accuracy) did not quite reach that of the gold‐standard Gleason score (79.67% accuracy) in the validation cohort. These data highlight the promise of this approach for aiding risk stratification, but the assay is open to further development to improve its prognostic suitability.

In summary, we have identified several mRNAs that are differentially expressed in EVs from disease‐associated stromal cells compared to those from normal stroma. We can detect some of these mRNAs in EVs from prostate cancer patient serum. We present a proof‐of‐concept assay whereby EV‐associated mRNAs can discriminate patients with high *vs* low Gleason score disease, acting as a surrogate measure of histological severity. Furthermore, a combined use of EV‐associated mRNAs together with PSA measurements informs a risk‐prediction model capable of identifying individuals most likely to exhibit rapid disease progression, and hence allowing prioritisation of men requiring clinical interventions.

## CONFLICT OF INTEREST

The authors declare no conflict of interest.

## Supporting information

Supporting Information

Supporting Information
