# Supplementary figures and images for: Stroma‐derived extracellular vesicle mRNA signatures inform histological nature of prostate cancer
Source: J Extracell Vesicles. 2021 Oct 1;10(12):e12150. doi: 10.1002/jev2.12150 (PMC8485336; doi:10.1002/jev2.12150)

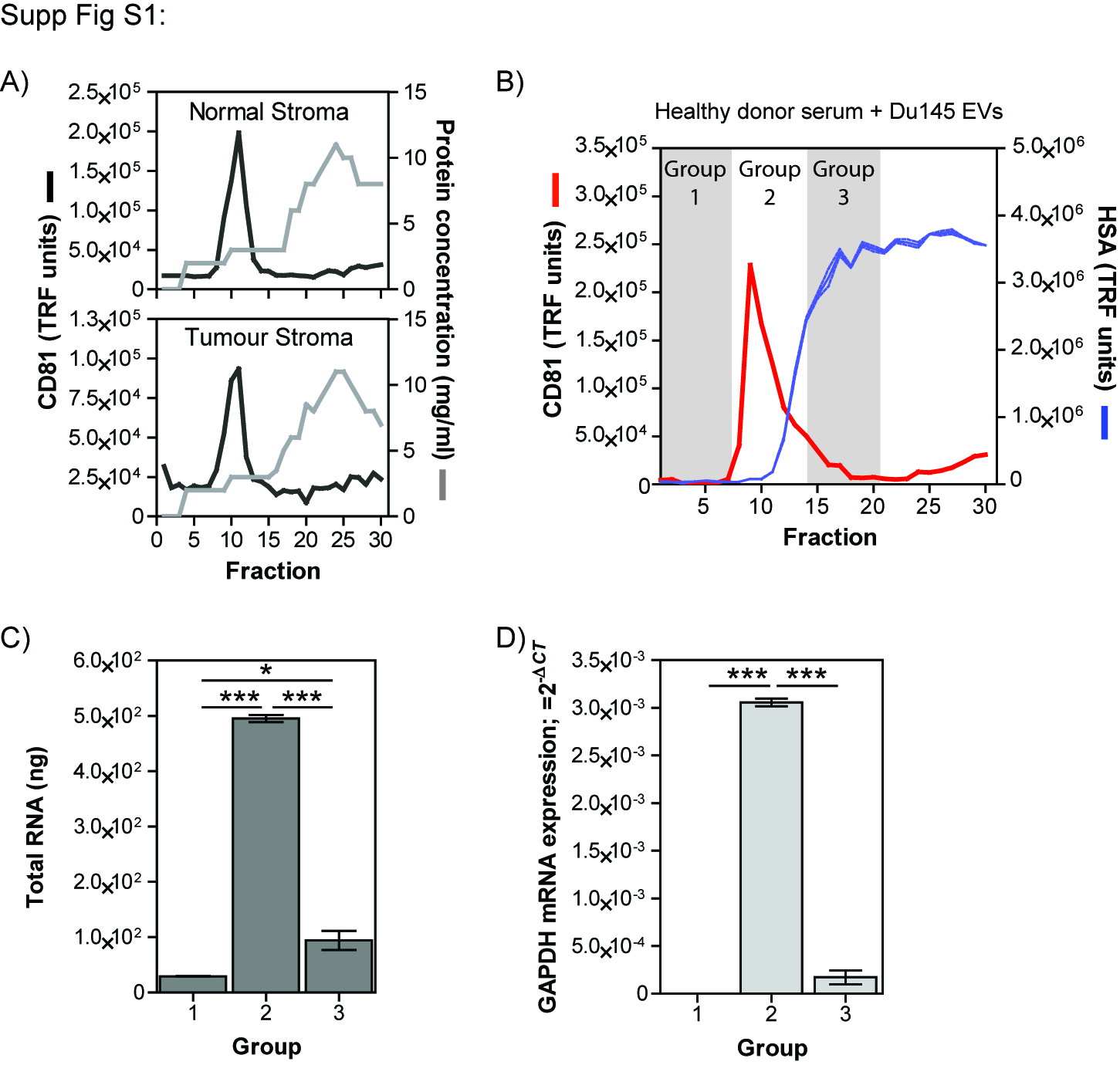

Supplement: Supplementary file 1 — Supporting Information [file JEV2-10-e12150-s001.tif]

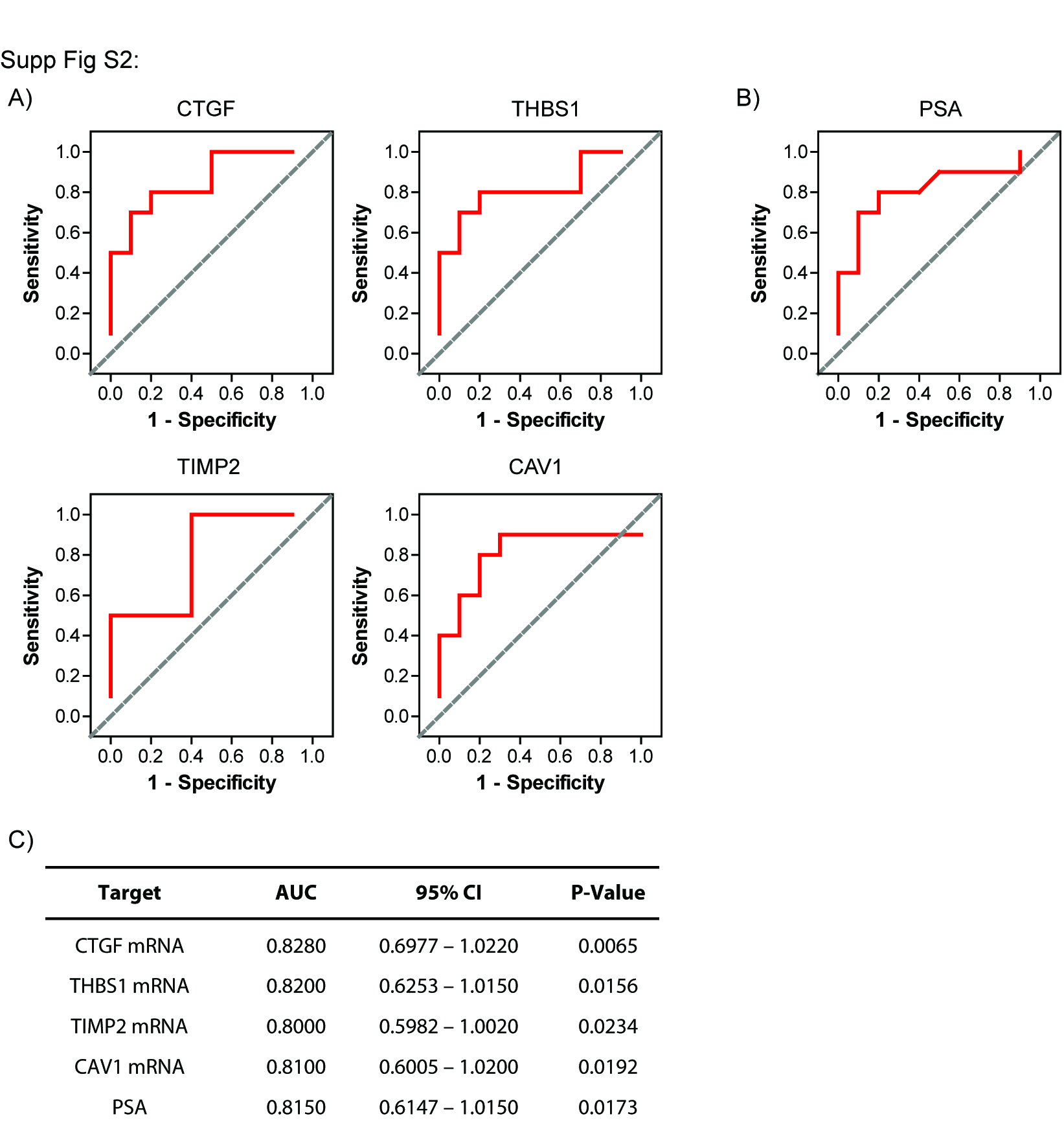

Supplement: Supplementary file 2 — Supporting Information [file JEV2-10-e12150-s002.tif]
